# Anti-tumor activity of nanomicelles encapsulating CXCR4 peptide antagonist E5

**DOI:** 10.1371/journal.pone.0182697

**Published:** 2017-08-09

**Authors:** Xiaocui Fang, Hanyi Xie, Hongyang Duan, Ping Li, Maryam Yousaf, Haiyan Xu, Yanlian Yang, Chen Wang

**Affiliations:** 1 CAS Key Laboratory of Standardization and Measurement for Nanotechnology, CAS Center for Excellence in Nanoscience, National Center for Nanoscience and Technology, Beijing, P. R. China; 2 CAS Key Laboratory of Biological Effects of Nanomaterials and Nanosafety, CAS Center for Excellence in Nanoscience, National Center for Nanoscience and Technology, Beijing, P. R. China; 3 Institute of Basic Medical Sciences, Chinese Academy of Medical Sciences & Peking Union Medical College, Beijing, P. R. China; University of South Alabama Mitchell Cancer Institute, UNITED STATES

## Abstract

Cancer is the leading cause of death worldwide, and metastasis is the main attribute to cancer death. CXCR4 and its natural ligand CXCL12 have been known to play a critical role in tumorigenesis, angiogenesis and metastasis. Therefore, designing a new CXCR4 antagonist to prevent tumor metastasis will be of great significance. Herein, a novel chemically synthesized peptide (E5) that has an ability to target CXCR4/CXCL12 axis was loaded in micelle glycol-phosphatidylethanolamine (PEG-PE) block copolymer to form micelle-encapsulated E5 (M-E5). We demonstrated that M-E5 exhibited higher affinity for CXCR4-overexpressing MCF-7 and HepG2 tumor cells as compared to free E5, and efficiently inhibited the tumor cells migration. Mechanistic studies implied that PEG-PE micelle can encapsulate E5 and improve E5 targeting efficiency for CXCR4 by accumulating E5 on the tumor cell membrane. Furthermore, through encapsulation of chemotherapeutic drug doxorubicin (Dox) in PEG-PE micelle, we proved that PEG-PE micelle could serve as a co-carrier for both E5 and Dox (M-E5-Dox). M-E5 enhanced the efficiency of Dox by down-regulating the phosphorylation level of Akt, Erk and p38/MAPK proteins. In conclusion, PEG-PE micelle demonstrated a promising delivery system for E5, and M-E5 is expected to be a potential therapeutic agent that will help to improve the clinical benefits in current therapies used for solid tumors.

## Introduction

Tumor metastasis is one of the leading cause of death in 90% of patients suffering from malignant tumor [1,2]. Mechanistically metastasis can be well described in two-phase, tumor metastasis starts with the migration of tumor cells from primary tumor to a distant organ of potential metastasis (first phase). Later on tumor cells proliferate within a micrometastasis that leads to the formation of a macroscopic metastatic lesion at the distant site (second phase) [3]. According to the seed-and-soil hypothesis of metastatic dissemination, the distant organ of potential tumor metastasis is not only determined by the characteristics of primary tumor cells (the ‘seed’), but also by the microenvironment in specific organs (the ‘soil’) that supports tumor cells adhesion and subsequent growth and proliferation [4–6].

Literature survey revealed that the diverse network of chemokines and their receptors play a critical role in primary tumor progression, angiogenesis and metastasis [7–9]. To date, it has been well-documented that the interaction between chemokine receptor 4 (CXCR4) and its ligand, stromal cell derived factor-1 (SDF-1, also termed as CXCL12), is closely associated with tumor cells adhesion, invasion and migration [7]. CXCR4/CXCL12 axis triggers PI3K-Akt and Ras-Erk signalling pathways, which mediates tumor cells survival and proliferation [8]. In addition, CXCR4 promotes the secretion of matrix metalloproteinases (MMPs), such as MMP2 and MMP9, which leads to the degradation of extracellular matrix (ECM) and facilitates the tumor cell motility [10]. In context of above mentioned factors, CXCR4/CXCL12 axis is widely accepted as a potential therapeutic target for cancer therapy. Until now, several promising CXCR4 antagonists have been developed to block CXCR4/CXCL12 axis that are still under different stages of development [11]. Currently, only one CXCR4 commercial antagonist, plerixafor (also termed as AMD3100), has been approved by the Food and Drug Administration (FDA) in 2008 for hematopoietic stem cell mobilization as an injectable agent for short-term treatment, while the long-term safety data for AMD3100 has not been available.

Due to the importance of CXCR4 in tumor cells invasion and migration, it is highly desirable to develop a new CXCR4 antagonist with high efficacy and low toxicity for tumor therapy. Bio-active peptides could be the therapeutic material of choice due to their low toxicity, high specificity, and significant progress in the solid-phase peptides synthesis technology during past few years. The number of peptides as FDA approved drugs and drug candidates has increased significantly in recent years [12–15]. In our previous studies, a novel synthetic peptide (E5) has been reported to interfere with CXCR4/CXCL12 axis. E5 significantly improved the therapeutic efficiency of various chemotherapeutics on acute myeloid leukemia (AML) *in vitro* and *in vivo* by diminishing the protection provided by bone marrow stromal cell [16,17].

Emerging evidence demonstrates that CXCR4 is not only overexpressed in AML but also in various types of human solid tumors, such as breast tumor, prostate tumor, lung tumor, melanoma tumor, and ovarian tumor [18]. Inspired by the significant inhibitory effect of E5 on AML, we applied E5 on overexpressed-CXCR4 solid tumor cells, and investigated whether E5 could sensitize tumor cells to chemotherapeutics. In order to increase the stability and bioactivity of E5, we developed poly(ethylene glycol)-phosphatidylethanolamine (PEG-PE) micelle-encapsulated E5 (M-E5) by a one-step self-assembly method. Extensive studies have shown that PEG-PE micelle is a promising nano-sized system for improving hydrophobic drug stability and anti-tumor activity both *in vivo* and *in vitro* [19–23]. Our studies showed that, encapsulating E5 in PEG-PE micelle could enhance the binding ability of E5 for CXCR4-overexpressing tumor cells that prevent the tumor cells motility. Furthermore, through encapsulation of chemotherapeutic drug doxorubicin (Dox) into M-E5, we found that M-E5 was capable of sensitizing tumor cells for Dox *in vitro* via blocking CXCR4/CXCL12 axis.

## Materials and methods

### Chemicals and reagents

Lyophilized FITC-labeled E5 and unlabeled E5 were purchased from GL Biochem Ltd. (Shanghai, China). PE mouse anti-human CXCR4 and PE mouse IgG2a (κ Isotype control) antibodies were purchased from BD Biosciences (San Jose, CA). CXCR4 primary antibody was obtained from Abcam (Cambridge, MA). The primary antibodies of Erk, phosphorylated-Erk, Akt, phosphorylated-Akt, p38, phosphorylated-p38 and GAPDH were purchased from Cell Signaling Technology Inc. (Beverly, MA). Protease inhibitor and phosphatase inhibitor cocktail 3 were purchased from Sigma-Aldrich Co. (Germany). Cell lysis buffer was purchased from Cell Signaling Technology Inc. (Beverly, MA). 1,2-distearoyl-sn-glycero-3-phosphoethanolamine-N-[methoxy(polyethyleneglycol)-2000] (PEG-PE), CXCL12 (SDF-1), and AMD3100 (plerixafor) were purchased from Avanti Polar Lipids (Alabama, USA), R&D Systems (Minneapolis, MN) and Selleckchem (Houston, USA), respectively. Doxorubicin hydrochloride (Dox) was a kind gift from Prof. Wei Liang in Institute of Biophysics. All other chemicals were of analytical grade and used without any further purification.

### Cell culture

Human breast tumor cell lines (MCF-7 and SKBR-3), hepatic carcinoma cell line (HepG2), pancreatic tumor cell line (Panc-1), prostate tumor cell line (PC-3), and cervical tumor cell line (Hela) were purchased from the Cell Resource Center of Chinese Academy of Medical Sciences (Beijing, China). All cells were cultured in a humidified atmosphere containing 5% CO_2_ at 37°C.

### Preparation of PEG-PE micelle, M-E5 and M-E5-Dox

PEG-PE micelles were prepared by dissolving PEG-PE powder in Milli-Q water, whereas E5-loaded micelles (M-E5) and E5/Dox-loaded micelles (M-E5-Dox) were prepared by a one-step self-assembly method. Briefly, for the preparation of M-E5, FITC labeled and unlabeled E5 and PEG-PE were first dissolved in Milli-Q water separately. Later on both solutions were mixed at different molar ratios (E5: PEG-PE ratio was varied from 1: 0 to 1: 5) and incubated at 55°C for 30 min. While for the preparation of M-E5-Dox, Dox was dissolved in Milli-Q water followed by mixing and incubation (55°C for 30 min) with M-E5 at different molar ratios (“E5: PEG-PE: Dox” was varied from 1: 200: 20 to 10: 10: 2). The reaction mixtures were diluted to the required concentration of 1X phosphate-buffered saline (PBS; pH 7.4) with 10X PBS for further investigation.

### Characterization of PEG-PE micelle, M-E5 and M-E5-Dox

Particle size distribution and zeta-potential of the empty PEG-PE micelles (50 μM), M-E5 (PEG-PE: 50 μM, E5: 12.5 μM) and M-E5-Dox (PEG-PE: 50 μM, E5: 12.5 μM, and Dox: 5 μM) were determined by dynamic light scattering (DLS) analysis using Nano Particle Analyzer (Malvern Instrument Ltd, Malvern, UK). The morphology of the empty PEG-PE micelle, M-E5 and M-E5-Dox was observed via TEM (Hitachi, Ltd., Tokyo, Japan) with 80 kV acceleration voltage.

### Drug encapsulation efficiency

Drug encapsulation efficiency of E5 and Dox was determined by ultrafiltration. Briefly, 500 μL of M-E5 (PEG-PE: 100 μM, FITC-E5: 25 μM) and M-E5-Dox (PEG-PE: 100 μM, FITC-E5: 25 μM, and Dox: 10 μM) were added in amicon ultra centrifugal filter devices (MWCO 100,000, Millipore), and then centrifuged at 12000 × g for 15 min at 4°C. The concentration of free Dox in filtrate was quantified by reverse-phase HPLC (RP-HPLC, Agilent 1206, USA) with a C_18_ column (ZORBAX 300SB-C18, 5 μm, 4.6 × 150 mm, Agilent, USA). The detection wavelength used was 254 nm, and the mobile phase was composed of sodium dodecyl sulfate (SDS) solution (2.88 g of SDS and 1.36 mL of phosphoric acid were co-dissolved in 1000 ml of Milli-Q water)-acetonitrile-methanol (25: 25: 3, v/v/v) at a flow rate of 1.0 ml/min, as previously reported [20]. The concentration of free E5 in filtrate was quantified using spectrophotometer (SpectraMax i3, Molecular Devices, USA). The encapsulation efficiency of Dox (E5) was calculated using Eq (1)

$$\%\;Encapsulation\;efficiency\;of\;Dox\;(E5)=\frac{\left[incorporated\;Dox\;(E5)\right]}{\left[initial\;Dox\;(E5)\;added\right]}*100$$ (1)

### *In vitro* drug release

*In vitro* release of Dox and E5 from M-E5-Dox (PEG-PE: 100 μM, FITC-E5: 25 μM, and Dox: 10 μM) was determined by dialysis using a 100,000 Da molecular weight cut-off membrane (Millipore). 5 mL of M-E5-Dox was dialyzed against PBS buffer (pH 7.4 and pH 5.0) at 37°C with gentle shaking. After 1, 2, 3, 4, 8, 12, 24, 48 and 72 h, 0.5 mL aliquot of PBS was withdrawn, and replaced with 0.5 mL of fresh PBS solution. The Dox and E5 concentrations were determined by RP-HPLC and spectrophotometer according to the above-mentioned experimental procedure. Accumulative release of Dox and E5 from M-E5-Dox were expressed as a percentage of the released Dox and E5 and plotted as a function of time.

### *In vitro* affinity assays of E5 and M-E5

Binding affinities of E5 and M-E5 for MCF-7 and HepG2 tumor cells were determined through concentration- and time-effect assay. Briefly, in the concentration-effect assay; tumor cells were incubated with FITC-E5 and M-E5 at different concentrations (FITC-E5: 0–10 μM, PEG-PE: 20 μM) for 2 h at 37°C. In the time-effect assay; tumor cells were incubated with FITC-E5 (5 μM) and M-E5 (FITC-E5: 5 μM, PEG-PE: 20 μM) at 37°C for different incubation times (0–5 h). 1×10^4^ cells were collected and subjected to Accuri^TM^ C6 flow cytometer (BD Biosciences, San Jose, CA), acquired data was analysed with CellQuest software.

In addition, the binding affinities of E5 and M-E5 for MCF-7 and HepG2 tumor cells were also determined by immunofluorescence using confocal microscope (LSM 700, Carl Zeiss, Germany). Tumor cells were cultured in opti-MEM medium (Life Technologies, GrandIsland, NY) for 30 min, blocked with 2% BSA for 30 min, and then incubated with FITC-E5 (5 μM) and M-E5 (FITC-E5: 5 μM, PEG-PE: 20 μM) for 2 h at 37°C.

### *In vitro* specificity assay

MCF-7 and HepG2 tumor cells were cultured in opti-MEM medium for 30 min, blocked with 2% BSA for 30 min at 37°C, and then pre-incubated with an excess of anti-CXCR4 primary antibody (1:50) for 30 min in serum-free medium. After washing with PBS, cells were incubated with M-E5 (FITC-E5: 5 μM, PEG-PE: 20 μM) for 1 h at 37°C, then fixed with 4% cold paraformaldehyde for 15 min at room temperature and stained with DAPI (0.1 μg/ml) for 10 min. FITC-E5 binding was visualized using confocal microscopy.

### Transwell assay

To assess the migration mediated by CXCL12, MCF-7 and HepG2 tumor cells were pre-treated with AMD3100 (5 μM, positive control), E5 (5 μM), and M-E5 (E5: 5 μM, PEG-PE: 20 μM) in opti-MEM medium at 37°C for 1 h and then seeded into the upper chambers of the transwell inserts (pore size is 8 μm, Millipore, Switzerland). 800 μL of complete DMEM medium with and without 200 ng/mL of CXCL12 (SDF-1) were added into the lower chambers. After 24 h of incubation, the non-invaded cells on the inserts were removed by wiping with a cotton swab whereas the invaded cells adhering to the bottom side of the inserts were fixed with 4% paraformaldehyde for 10 min. After that, the inserts were stained with 0.1% crystal violet solution (HBK Pharmaceutical Technology Co., Beijing, China) for 30 min. Later on images of stained cells were taken by EVOS microscope, and five 200X imaging areas were randomly selected for each insert. In addition, the stained cells were dissolved in 33% acetic acid for 10 min, and the absorbance of the solution was measured at 570 nm using spectrophotometer. When CXCL12 (200 ng/mL) was supplemented in the lower chamber of the transwell insert, then the invaded cells without any treatment adhering to the bottom side of the insert were served as a control. The percentage of migrated cells was calculated using Eq (2)

$$\%\;Migrated\;cells\;=\;\frac{sample}{control}*100$$ (2)

### Real-time reverse transcription-polymerase chain reaction (RT-PCR)

To address the effect of E5 on the mRNA expression level of the EMT markers in the absence and presence of PEG-PE micelle, MCF-7 and HepG2 tumor cells were pre-treated with E5 (5 μM) and M-E5 (E5: 5 μM, PEG-PE: 20 μM) for 1 h, followed by treatment with CXCL12 (200 ng/ml) for another 30 min. After that, total RNAs were isolated from cells using Trizol. The mRNAs were reversely transcribed to cDNAs by QuantScript RT Kit. The mRNA expression level was determined using Super Real PreMix Plus (SYBR Green) according to the instrument: PCR was initiated by a 15 min denaturation at 95°C, followed by 40 cycles of 95°C for 10 s, 58°C for 20 s, and 72°C for 30 s using Realplex4 Detection System (eppendorf, Germany). The mRNA expression level was normalized to GAPDH (a housekeeping gene) and presented as a fold-change compared to the control experiments for all samples, as previously described [24]. Cells without any treatment served as a control.

### Western blot analysis

MCF-7 and HepG2 tumor cells were cultured overnight in opti-MEM medium and pre-treated with E5 (5 μM) and M-E5 (E5: 5 μM, PEG-PE: 20 μM) for 1 h at 37°C, followed by treatment with CXCL12 (200 ng/ml) for another 30 min. Later on, cells were harvested, washed, and lysed in ice cold RIPA lysis buffer containing a mixture of protease inhibitor and phosphatase inhibitor for 30 min. The debris was removed by centrifugation at 14,000 x g for 10 min. Equal amount of proteins (20 μg) from each sample was loaded and separated on 10% polyacrylamide gel and transferred to a PVDF membrane (0.45 μm; Millipore, Bedford, MA) by semidry transfer (Trans-Blot SD Semi-dry Transfer cell, Bio-Rad, USA). The membrane was blocked with 5% (w/v) BSA in Tris-buffered saline containing 0.1% Tween 20 (TBST) for 1 h at room temperature. Later on the membrane was treated with primary antibodies (Erk, phosphorylated-Erk, Akt, phosphorylated-Akt, p38, phosphorylated-p38, GAPDH) overnight at 4°C followed by incubation with the appropriate peroxidase-conjugated secondary antibody for 1 h at room temperature. The membrane was washed with TBST for three times and then developed with enhanced chemiluminescence system.

### Caspase-3 activity assay

Caspase-3 activity assay quantifies caspase-3 activation *in vitro* by measuring the cleavage of caspase-3 substrate DEVD-7-amino-4-trifluoromethyl coumarin (AFC) to free AFC, which emits yellow-green fluorescence (λ_max_ = 505 nm) [25]. MCF-7 and HepG2 tumor cells were cultured with free Dox (2 μM), M-Dox (Dox: 2 μM, PEG-PE: 20 μM) and M-E5-Dox (Dox: 2 μM, PEG-PE: 20 μM, and E5: 5 μM) for 24 h at 37°C. After that, cells were washed and treated with lysis buffer, and 50 μL of supernatant was transferred to 96-well plate followed by the addition of 50 μL of reaction buffer to each sample well. The free AFC is measured at an excitation wavelength of 415 nm and an emission wavelength of 505 nm using spectrophotometer. Cells without any treatment were served as a control. Caspase-3 activity of each sample was normalized by cellular protein mass (BCA^TM^ Protein Assay Kit) and was calculated using Eq (3)

$$\%\;Caspase-3\;activity\;=\;\left[\frac{Sample\;fluorescence}{Total\;sample\;lysate\;protein}\right]÷\left[\frac{Mean\;of\;media\;control\;fluorescence}{Mean\;of\;total\;media\;control\;lysate\;protein}\right]*100$$ (3)

#### Cell viability assay

The effects of various combinations of drugs on the viabilities of MCF-7 and HepG2 tumor cells were measured using the CellTiter 96® Aqueous One Solution Cell Proliferation assay (also termed as MTS assay; Promega, Madison, Wisconsin, USA) according to the manufacturer’s protocol. Briefly, 2×10^4^ of tumor cells were plated in 96-well plates and grown for 24 h. Then the cells were incubated with a series of Dox concentrations (0–2 μM) combined with PEG-PE (M-Dox, PEG-PE: 20 μM), E5 (E5 + Dox, E5: 5 μM), M-E5 (M-E5-Dox, PEG-PE: 20 μM, E5: 5 μM) and AMD3100 (AMD3100 + Dox, AMD3100: 5 μM) for 48 h at 37°C. After being washed with PBS, 20 μL of MTS reagent was added to each well and incubated for another 2 h. The absorbance was measured at 490 nm using spectrophotometer. Wells with culture medium alone (no cells) served as blank controls, and the value of cells without any treatment was set as 100%. In addition, the cytotoxicity assays of PEG-PE (0–200 μM), E5 (0–100 μM) and AMD3100 (0–200 μM) were also quantified according to the above-mentioned method.

### Statistical analysis

All experiments were carried out at least three times with three independent samples. Data were expressed as means ± SD unless noted otherwise. Unpaired Student’s t-test was performed to assess the statistical significance of the results and was expressed in terms of p values, p value less than 0.05 are indicated by * and p value less than 0.01 are indicated by **.

## Results and discussion

### Preparation, characterization and drug release of M-E5 and M-E5-Dox *in vitro*

**Fig 1:**
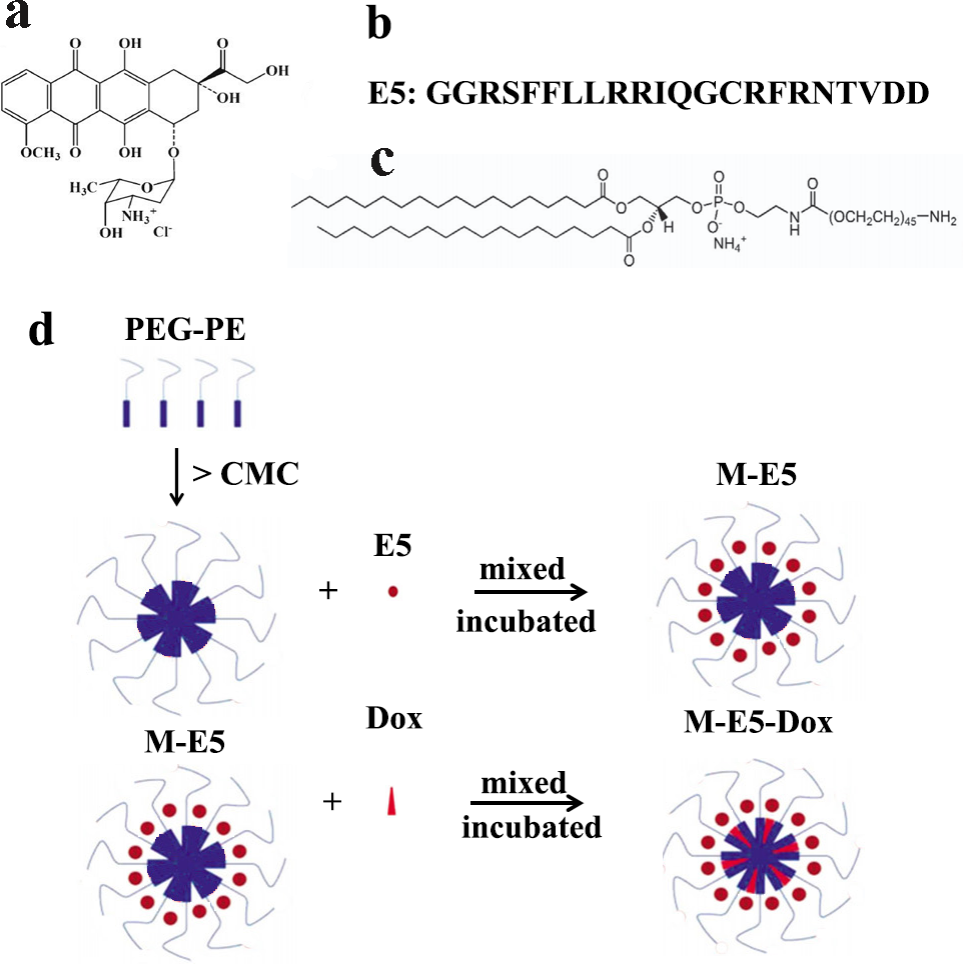
Preparation of drug-loaded PEG-PE micelles. Chemical structures of (**a**) doxorubicin hydrochloride, (**b**) E5 peptide sequence (from N to C), and (**c**) PEG-PE. (**d**) Schematic representation of drug-encapsulation of PEG-PE micelles (M-E5 and M-E5-Dox) by a one-step self-assembly method.

In our previous studies, we have shown that a number of water-soluble drugs, such as Dox, vinorelbine tartrate and epirubicin hydrochloride, can be effectively incorporated into PEG-PE micelles with high drug-loading efficiency (>99.0%) using a one-step self-assembly method [20,26,27]. This method is very simple, fast and efficient for preparing the drug carriers in aqueous solution without using organic solvents. In this study, we have investigated the interaction of two water-soluble anticancer drugs E5 and Dox (both are composed of hydrophobic and positively charged groups) with PEG-PE micelles. Fig 1 illustrated a one-step self-assembly method of E5 encapsulation and E5/Dox co-encapsulation in PEG-PE micelle to form M-E5 and M-E5-Dox, respectively. This PEG-PE polymer is negatively charged at physiological pH due to the de-protonation of the phosphate group that exists between hydrophilic PEG and hydrophobic PE. Dox and E5 can bind with the phosphate group of PEG-PE and distribute over the amphiphilic core-shell interface of PEG-PE micelles. The electrostatic and hydrophobic interactions between these two drugs and PEG-PE were found to play a critical role in high drug encapsulation efficiency and controlled drug release, in agreement with the previous studies [19,20].

**Fig 2:**
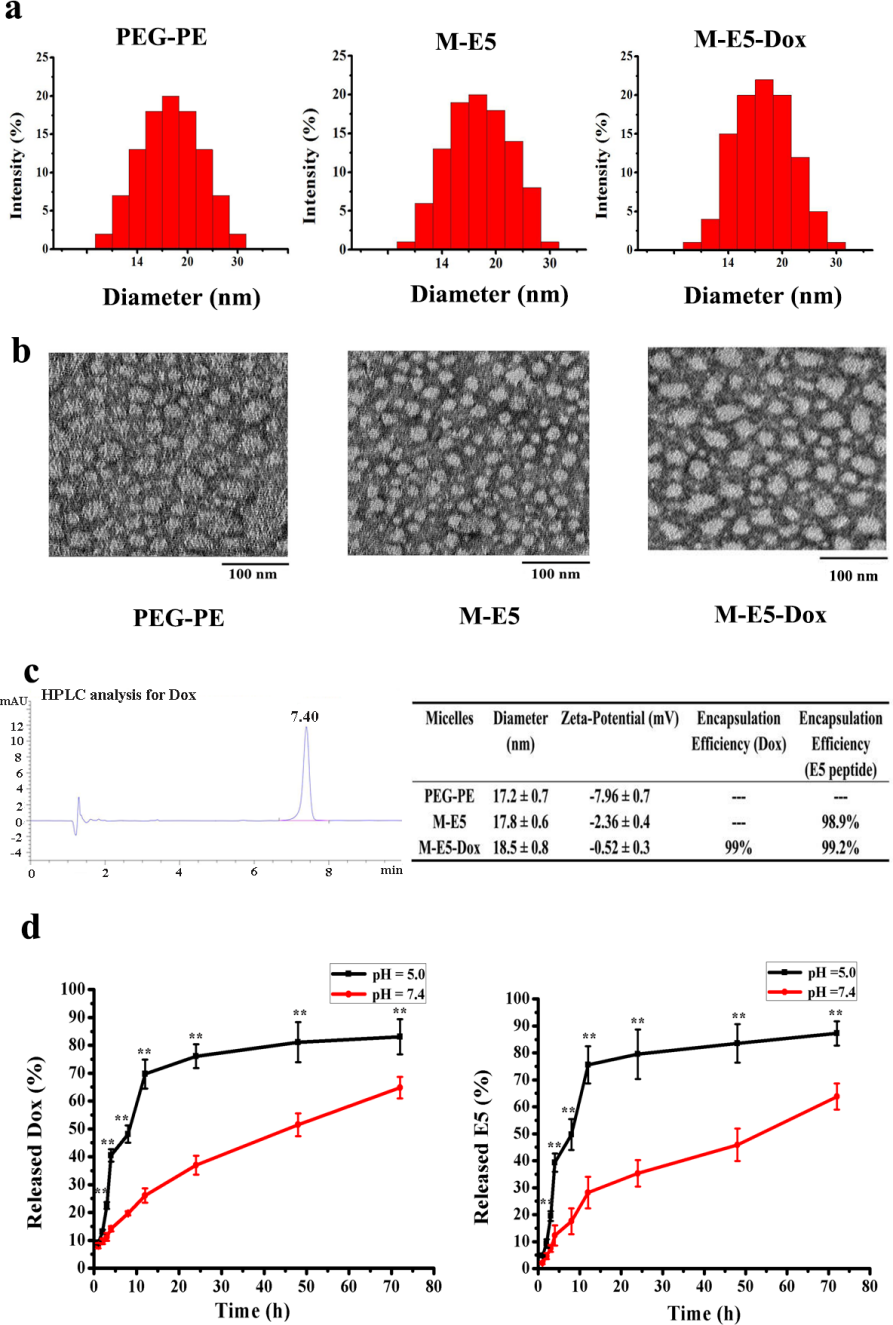
Characterization of micelles in terms of particle size distribution, morphology and drug release kinetics. **(a**) Particle sizes of the PEG-PE micelle (50 μM), M-E5 (PEG-PE: 50 μM, E5: 12.5 μM) and M-E5-Dox (PEG-PE: 50 μM, E5: 12.5 μM, and Dox: 5 μM) were determined by DLS. (**b**) The morphology of the PEG-PE micelle (50 μM), M-E5 (PEG-PE: 50 μM, E5: 12.5 μM) and M-E5-Dox (PEG-PE: 50 μM, E5: 12.5 μM, and Dox: 5 μM) was observed via TEM, and the samples were stained with 1% uranyl acetate for 1 min at room temperature. Scale bar = 100 nm. (**c**) The HPLC chromatogram of Dox, and physicochemical properties of the PEG-PE micelle (100 μM), M-E5 (PEG-PE: 100 μM, E5: 25 μM) and M-E5-Dox (PEG-PE: 100 μM, E5: 25 μM, and Dox: 10 μM). (**d**) Time course (0–72 h) release profiles of Dox and E5 from M-E5-Dox (PEG-PE: 100 μM, E5: 25 μM, and Dox: 10 μM) at 37°C and at pH 5.0 and 7.4. Data are presented as mean ± SD (*n* = 3). The * represents significant difference between two groups (*p < 0.05, **p < 0.01).

The average hydration radius and surface charge of the empty PEG-PE micelle, M-E5, and M-E5-Dox were characterized by DLS, as shown in Fig 2A and 2C. The diameter of the empty PEG-PE micelle was 17.2 ± 0.7 nm with observed zeta-potential of -7.96 ± 0.7 mV, consistent with previous studies [26]. The sizes of M-E5 at a molar ratio of 4: 1 (PEG-PE: E5) and M-E5-Dox at a molar ratio of 20: 5: 2 (PEG-PE: E5: Dox) were similar, with diameters of 17.8 ± 0.6 nm and 18.5 ± 0.8 nm, respectively. Meanwhile, the morphology of micelles observed by TEM showed that incorporation of E5 and Dox did not disrupt the structure of PEG-PE micelles. All three prepared micelles were spherical with a uniform size of about 20 nm, consistent with the DLS results (Fig 2B).

In addition, we examined the efficiency of PEG-PE micelle to encapsulate Dox and E5 using dialysis method and quantified by RP-HPLC and spectrophotometer. As shown in Fig 2C, the run time of the RP-HPLC analysis was 10 min, and the retention time obtained for Dox was 7.40 min. PEG-PE micelle showed a high encapsulation efficiency for both E5 and Dox, and E5 incorporation did not affect Dox loading efficiency *vice versa*. Dox encapsulation efficiency is 99% in M-E5-Dox at a molar ratio of 20: 5: 2 (PEG-PE: E5: Dox), whereas E5 encapsulation efficiency is 98.9% in M-E5 at a molar ratio of 4: 1 (PEG-PE: E5) and 99.2% in M-E5-Dox at a molar ratio of 20: 5: 2 (PEG-PE: E5: Dox). The empty PEG-PE micelle had negative zeta-potential of -2.36 ± 0.4 mV due to the de-protonation of the phosphate group. Loading cationic drugs increased micellar zeta-potential of M-E5 (-2.36 ± 0.4 mV) and M-E5-Dox (-0.52 ± 0.3 mV), implying that the electrostatic interaction between drug and PEG-PE plays an important role in drug loading.

Controlled and sustained drug release is very important for the development of good drug delivery systems and therapeutic effect. Drug release is affected by a number of factors, and one of them is the pH of surrounding tissues environment. The pH of solid tumor tissues is much lower than that of normal tissues due to hypoxia and acidic intracellular organelles [28,29]. Therefore, the release profiles of Dox and E5 from M-E5-Dox (PEG-PE: 100 μM, FITC-E5: 25 μM, and Dox: 10 μM) were evaluated at pH 5.0 and 7.4 using dialysis method. From Fig 2D, it is obvious that either E5 or Dox release was much lower at pH 7.4 than at pH 5.0. At pH 7.4, Dox and E5 were slowly released from M-E5-Dox without initial burst release, 64.8% of Dox and 63.8% of E5 were released from M-E5-Dox up to 72 h. Meanwhile, at pH 5.0, about 83.1% of Dox and 87.2% of E5 were released up to 72 h. These pH-sensitive drug release profile could be related to the isoelectric points (PI) of Dox (PI: 9.06), E5 (PI: 11.4) and PEG-PE polymer (PI: 5.93). When the pH was reduced from 7.4 to 5.0, dissociation of Dox (or E5) was enhanced, and the electrostatic repulsion between drug molecules was increased. Moreover, the negatively charged PEG-PE molecules became positively charged and lost their electrostatic attraction for Dox and E5. From the above results, PEG-PE micelle exhibited different E5- and Dox-releasing profiles at pH 5.0 and 7.4, which may play an important role clinically in strengthening therapeutic effects on solid tumor tissues and in decreasing damage to normal tissues.

### M-E5 has high affinity for CXCR4 overexpressing human solid tumor cells

To evaluate the affinity of E5 in the absence and presence of PEG-PE micelle for tumor cells, firstly CXCR4 expression levels over the surface of six solid tumor cell lines (MCF-7, SKBR-3, HepG2, Panc-1, PC-3, and Hela) were measured by flow cytometry. From the result of panels a and b in S1 Fig, it could be clearly seen that MCF-7 (97.8%) and HepG2 (87.4%) tumor cells had higher CXCR4 expression levels as compared to SKBR-3 (5.9%), Panc-1 (0.5%), PC-3 (1.0%), and Hela (27.2%) tumor cells. In addition, the CXCR4 expression levels of MCF-7 and HepG2 tumor cells were also corroborated by immunofluorescence (Panel c in S1 Fig), implying that these two tumor cells overexpressed CXCR4 on their cell membranes.

**Fig 3:**
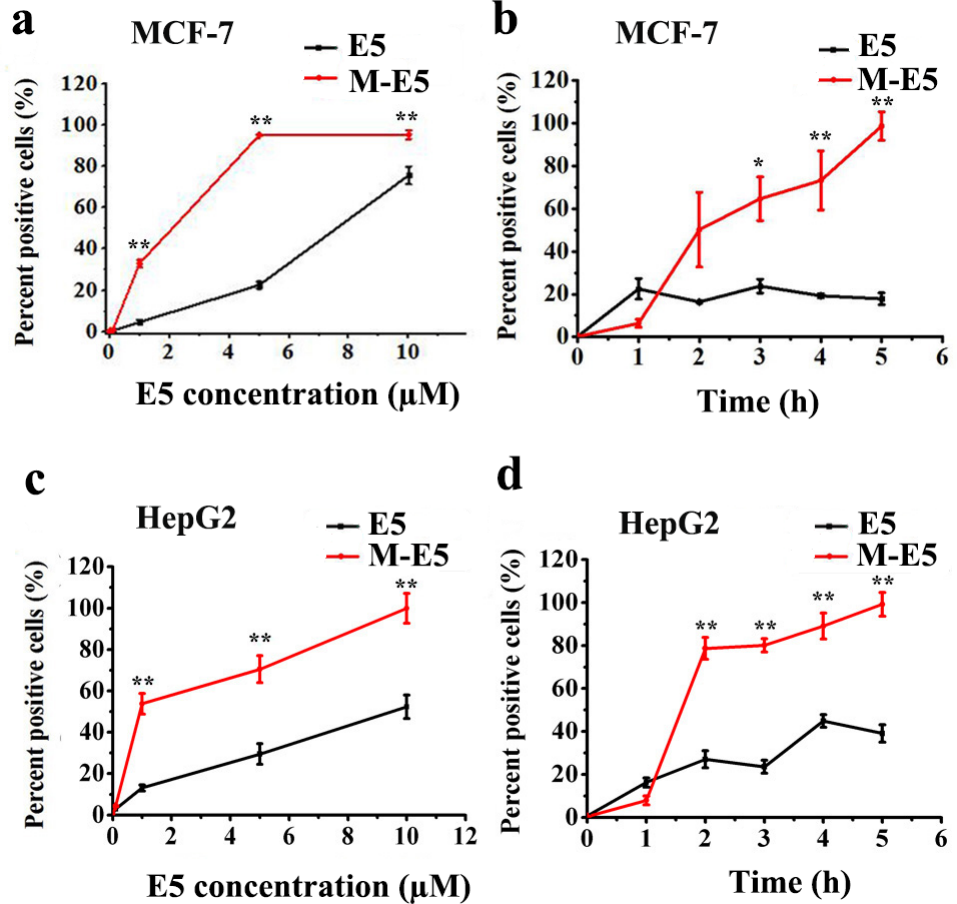
Binding assays of E5 in the absence and presence of PEG-PE micelles with tumor cells. Concentration-dependent binding ability of E5 (1–10 μM) for (**a**) MCF-7 and (**c**) HepG2 tumor cells after 2 h of incubation at 37°C, in the absence and presence of PEG-PE micelles (20 μM). Time course (0–5 h) binding ability of E5 (5 μM) for (**b**) MCF-7 and (**d**) HepG2 tumor cells, in the absence and presence of PEG-PE micelles (20 μM). Data are presented as mean ± SD (*n* = 3). The * represents significant difference between two groups (*p < 0.05, **p < 0.01).

Later on MCF-7 and HepG2 tumor cells were used to assess the CXCR4 targeting affinity of E5. Cells were incubated with FITC-E5 and M-E5 (FITC-E5: 1–10 μM, PEG-PE: 20 μM) for 0–5 h, and the affinity of E5 for cells was evaluated by flow cytometry. The binding amount of E5 depends on both incubation time and concentration, and the kinetic binding curves of E5 with MCF-7 and HepG2 tumor cells were similar (Fig 3). After 2 h of incubation, when the concentration of E5 was increased from 1 to 10 μM, the percentage fluorescence of M-E5 treated tumor cells was much higher than that of free E5 treated tumor cells. In addition, we also checked the effect of variation in time over the binding amount of E5 (5 μM) with tumor cells in the absence and presence of PEG-PE micelles (20 μM). The results indicated that during the initial 1.5 h, the percentage fluorescence of M-E5 treated tumor cells was less than that of free E5 treated tumor cells. A possible explanation for this phenomenon is that E5 was encapsulated by PEG-PE micelle and could not interact with CXCR4 in a very short time. After 1.5 h, PEG-PE copolymers is disassembled on the cell membrane and released E5 allowing the interaction of E5 with CXCR4. The percentages fluorescence of M-E5 treated MCF-7 and HepG2 tumor cells measured up to 5 h were 98.7% and 97.6%, respectively. In contrast, the percentages fluorescence of free E5 treated MCF-7 and HepG2 tumor cells were 17.9% and 23.7%, respectively.

**Fig 4:**
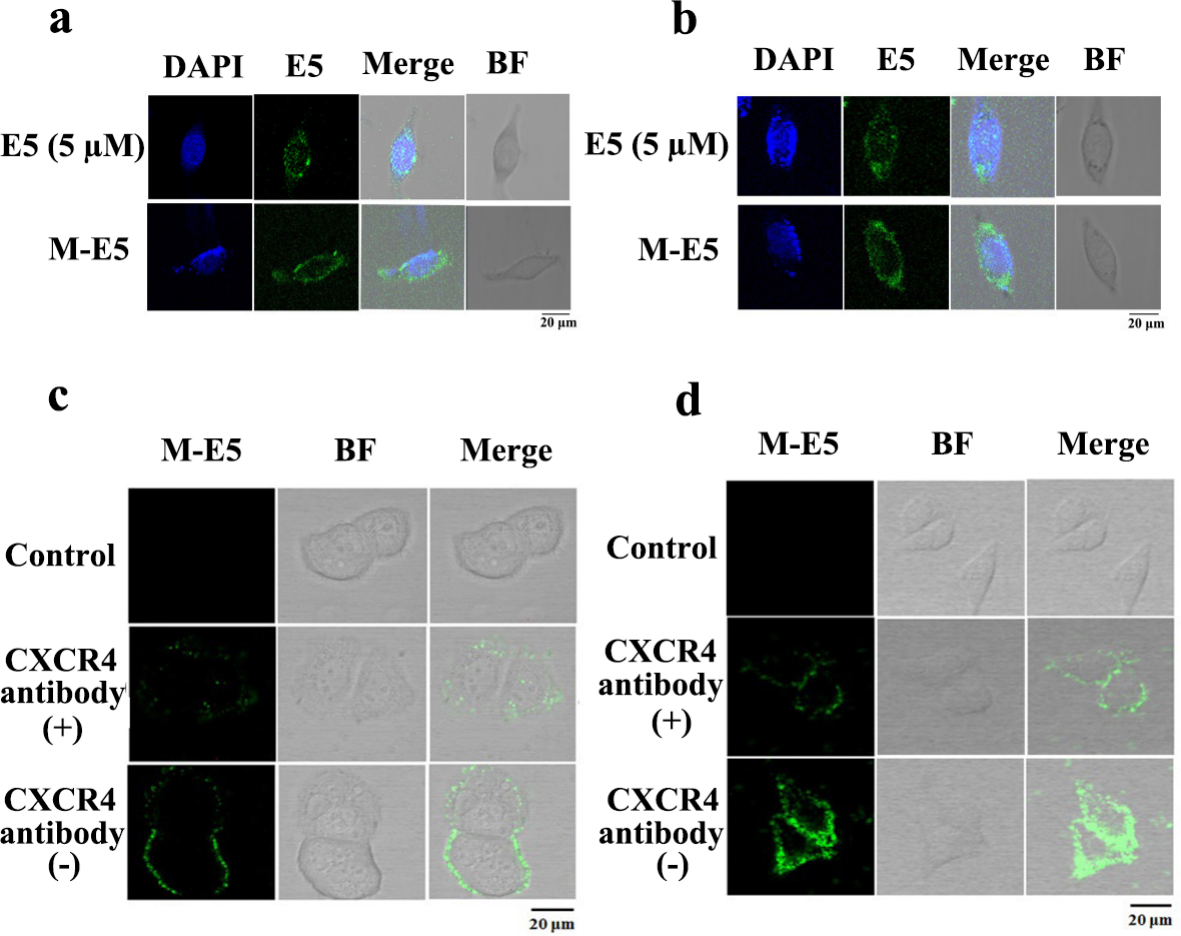
The binding affinity of M-E5 for CXCR4-overexpressing tumor cells. Confocal images of (**a**) MCF-7 and (**b**) HepG2 tumor cells after incubation with FITC-E5 (5 μM) in the absence and presence of PEG-PE micelles (20 μM) for 2 h at 37°C. *In vitro* M-E5 targeting specificity assays of (**c**) MCF-7 and (**d**) HepG2 tumor cells for CXCR4.

To further confirm higher binding affinity of M-E5 for MCF-7 and HepG2 tumor cells than that of free E5, an immunofluorescence assay was also carried out. As shown in Fig 4A and 4B, two tumor cell lines were incubated with FITC-E5 and M-E5 for 2 h, and the immunofluorescence results indicated that FITC-E5 effectively targeted CXCR4 in the presence of PEG-PE micelle, which was consistent with the flow cytometry results. In addition to MCF-7 and HepG2 cells, we also chose SKBR-3 cells as a negative control of low-expressed CXCR4 tumor cell line and assessed the affinities of E5 and M-E5 targeting CXCR4 by flow cytometry and immunofluorescence. For both E5- and M-E5-treated SKBR-3 cells, low percentage of fluorescent tumor cells (Panels a and b in S2 Fig) and no FITC-E5 fluorescence signal (Panel c in S2 Fig) were observed, demonstrating that E5 has no specific affinity for cells of low CXCR4 expression. E5 can exhibit its high activity via targeting over-expressed CXCR4 tumor cells, but due to relatively low CXCR4 expression level on the normal cells membrane, E5 does not show its effect. Furthermore, in order to evaluate the targeting specificity of M-E5 for CXCR4, MCF-7 and HepG2 tumor cells were capped with excess anti-CXCR4 primary antibody for 30 min followed by incubation with M-E5 at a molar ratio of 4: 1 (PEG-PE: 20 μM, FITC-E5: 5 μM) for 1 h at 37°C. The binding amount of M-E5 with CXCR4 antibody-treated cells was greatly inhibited as compared to untreated cells (Fig 4C and 4D). This result suggested that M-E5 was capable of specifically binding with CXCR4-overexpressing solid tumor cells.

### M-E5 inhibits CXCL12-induced migration of solid tumor cells

In recent years, a growing number of studies on CXCR4/CXCL12 axis have demonstrated that CXCL12 is highly secreted by regional lymph nodes, liver, lungs or bone marrow, and can stimulate CXCR4-overexpressing tumor cells migration [30]. On the other hand, epithelial-to-mesenchymal transition (EMT) plays an important role in primary tumor progression and metastasis, and this process is characterized by loss of epithelial and acquisition of mesenchymal characteristics of tumor cells [31–33]. During EMT progression, the expression levels of mesenchymal markers and matrix metalloproteinases (MMPs) are increased, in contrast, epithelial adhesion of molecules are decreased, as previously reported [24,34].

**Fig 5:**
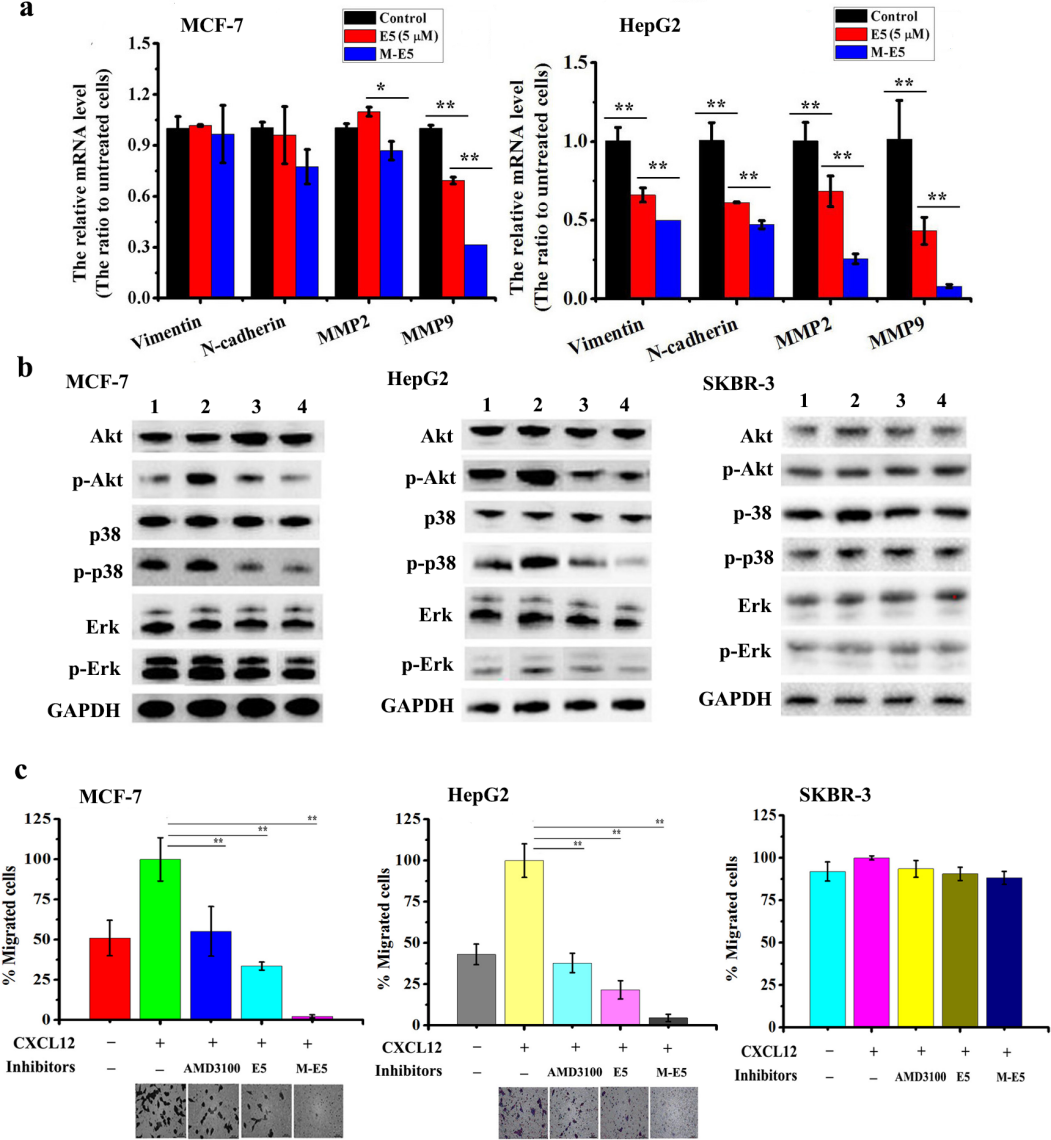
Effects of E5, M-E5 and AMD3100 on tumor cells migration. **(a**) mRNA expression level of EMT markers of MCF-7 and HepG2 tumor cells, measured by RT-PCR, after treatment with E5 (5 μM) in the absence and presence of PEG-PE micelles (20 μM). (**b**) Effects of E5 and M-E5 on CXCL12-induced phosphorylation level of Akt, p38 and Erk proteins of MCF-7, HepG2 and SKBR-3 tumor cells. *Lane 1*: control, *Lane 2*: CXCL12 treatment for 30 minutes at 200 ng/mL, *Lane 3*: treated with E5 at 5 μM for 1 h followed by CXCL12 treatment for 30 minutes at 200 ng/mL, *Lane 4*: treated with M-E5 (E5: 5 μM, PEG-PE: 20 μM) for 1 h followed by CXCL12 treatment for 30 minutes at 200 ng/mL. (**c**) Effects of E5, M-E5, and AMD3100 on CXCL12-induced migration of MCF-7, HepG2 and SKBR-3 tumor cells using transwell assays. Data are presented as mean ± SD (*n* = 3). The * represents significant difference between two groups (*p < 0.05, **p < 0.01).

In order to determine the effects of E5 in the absence and presence of PEG-PE micelle on the chemotaxis of MCF-7 and HepG2 tumor cells in response to CXCL12, real-time PCR (RT-PCR) analysis was carried out. The primers sequences of EMT markers are shown in S1 Table. Total RNA was isolated from the MCF-7 and HepG2 tumor cells that were pre-treated by E5 (5 μM) and M-E5 (E5: 5 μM, PEG-PE: 20 μM) for 1 h, followed by treatment with CXCL12 (200 ng/ml) for another 30 min. Our results indicated that, compared with untreated cells, the mRNA expression levels of EMT markers were significantly reduced by treatment with E5 and M-E5, such as vimentin, N-cadherin, MMP2 and MMP9 (Fig 5A). The mRNA expression levels of EMT markers appear to be decreased significantly after treatment with M-E5 compared with free E5.

The CXCR4/CXCL12 axis has been demonstrated to trigger PI3K-Akt and Ras-Erk signalling pathways, which accounts for the migration and adhesion of tumor cells conferred by CXCL12 [8,35]. To clarify either E5 or M-E5 inhibits tumor cells migration and adhesion through affecting the above-mentioned intracellular signalling pathways, western blot analysis was performed for evaluating protein expression level of Erk (phospho-Erk), Akt (phosphor-Akt) and p38/MAPK (phospho-p38) in MCF-7, HepG2 and SKBR-3 tumor cells. Cells were pre-treated with E5 and M-E5 for 1 h, followed by treatment with CXCL12 (200 ng/ml) for another 30 min. As shown in Fig 5B, as compared to untreated cells, the phosphorylation level of Erk, Akt and p38 were significantly decreased when MCF-7 and HepG2 tumor cells were pre-treated with E5 and M-E5. In contrast, Erk (phospho-Erk), Akt (phosphor-Akt) and p38/MAPK (phospho-p38) expression level of E5 and M-E5 treated SKBR-3 tumor cells showed no change as compared to control. These results clearly indicated that E5 could inhibit CXCR4-overexpressing tumor cells from responding to CXCL12 stimulation via blocking CXCR4/CXCL12 axis, and repress the CXCR4 downstream of Akt, Erk and p38/MAPK signalling pathways for tumor cell invasion and migration. It’s obvious from results that M-E5 showed better inhibitory effect than free E5.

Transwell assay was performed to evaluate whether the decreased mRNA expressions of vimentin, N-cadherin, MMP2 and MMP9, and the decreased phosphorylation level of p-Erk, p-Akt and p-p38/MAPK proteins for tumor cells are associated with their motility in response to CXCL12. When CXCL12 was supplemented in the lower chamber of the transwell inserts, tumor cells preferred to migrate into the chamber (set as 100% as the control) as compared to the tumor cells in the absence of CXCL12 in the lower chamber (MCF-7: 51%, HepG2: 43%, and SKBR-3: 92%). As shown in Fig 5C, E5 and M-E5 could inhibit the migration activity of MCF-7 and HepG2, but didn’t show the obvious inhibitory effect on SKBR-3 tumor cells, in agreement with PCR and western blot analysis. This result was corroborated by treatment with another CXCR4 antagonist, AMD3100. Taken together, these data demonstrated that E5 has a remarkable effect on interrupting the chemotactic response of CXCR4-overexpressing tumor cells to CXCL12. Due to the higher affinity of E5 for MCF-7 and HepG2 tumor cells in the presence of PEG-PE micelle (Figs 3 and 4), M-E5 has a greater impact on inhibiting CXCR4-mediated, CXCL12-induced tumor cells adhesion and migration than that of free E5.

### M-E5 enhances the sensitivity of solid tumor cells for chemotherapeutics *in vitro*

MTS assay was conducted to examine the cytotoxic effects of E5, PEG-PE and AMD3100 on MCF-7 and HepG2 tumor cells after 48 h treatment at 37°C. When E5 and AMD3100 concentrations were less than 100 and 200 μM, the viabilities of MCF-7 (Panels a and c in S4 Fig) and HepG2 tumor cells (Panels b and d in S4 Fig) were above 90%, which proved that E5 and AMD3100 were non-toxic to MCF-7 and HepG2 tumor cells at this concentration. Meanwhile, when the PEG-PE concentration was less than 100 μM, PEG-PE didn’t show obvious cytotoxicity to both MCF-7 (Panel e in S4 Fig) and HepG2 tumor cells (Panel f in S4 Fig). However, when the PEG-PE concentration was increased from 100 to 200 μM, the cells viabilities were dropped from 90% to 80%, suggesting the cytotoxicity to the solid tumor cells at high concentrations, which was consistent with the previous report [23]. Taking the cytotoxicity issue into consideration, we used E5, PEG-PE and AMD3100 concentrations under 20, 50 and 5 μM, respectively, for the following *in vitro* experiments.

**Fig 6:**
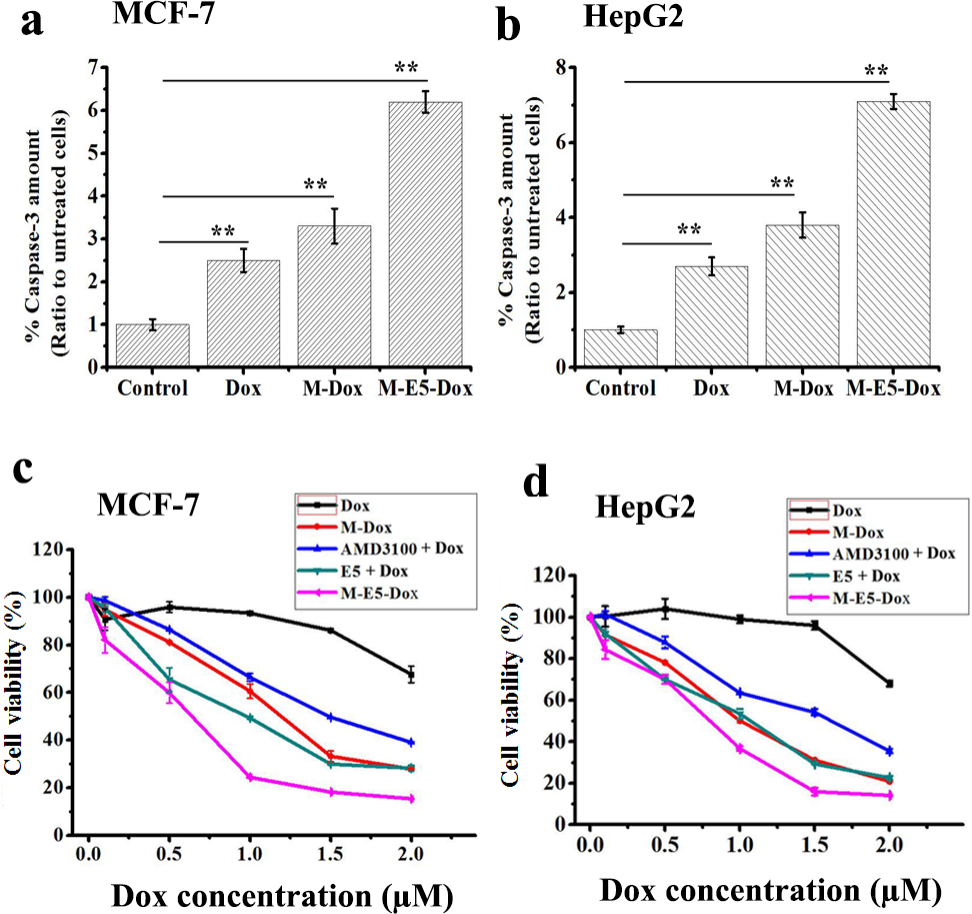
*In vitro* cytotoxicity assays of tumor cells. Effects of Dox (2 μM), M-Dox (Dox: 2 μM, PEG-PE: 20 μM) and M-E5-Dox (Dox: 2 μM, PEG-PE: 20 μM, and E5: 5 μM) on caspase-3 activities of **(a**) MCF-7 and (**b**) HepG2 tumor cells after 24 h treatment at 37°C. Cell viabilities of (**c**) MCF-7 and (**d**) HepG2 tumor cells, assessed by MTS, after incubation with free Dox, M-Dox, AMD3100 + Dox, E5 + Dox, and M-E5-Dox for 48 h at 37°C. Data are presented as mean ± SD (*n* = 3). The * represents significant difference between two groups (*p < 0.05, **p < 0.01).

To investigate whether E5 could increase the sensitivity of MCF-7 and HepG2 tumor cells for chemotherapeutic drugs, we combined PEG-PE micelle, AMD3100, E5 and M-E5 with Dox in the co-culture system. Apoptosis in mammalian cells is initiated by activation of the caspase family of cysteine proteases [23,36]. MCF-7 and HepG2 tumor cells were treated with Dox (2 μM), M-Dox (Dox: 2 μM, PEG-PE: 20 μM) and M-E5-Dox (Dox: 2 μM, PEG-PE: 20 μM, and E5: 5 μM) for 24 h at 37°C, followed by measurement of caspase-3 activities, as previously mentioned [25]. In MCF-7 tumor cells, the caspase-3 activities after free Dox, M-Dox and M-E5-Dox treatments were increased by 2.5 ± 0.3, 3.3 ± 0.4 and 6.2 ± 0.3 folds as compared to the control, respectively (Fig 6A). Meanwhile, in HepG2 tumor cells, the caspase-3 activities after free Dox, M-Dox and M-E5-Dox treatments were increased by 2.7 ± 0.2, 3.8 ± 0.3 and 7.1 ± 0.2 folds compared with untreated cells, respectively (Fig 6B).

**Fig 7:**
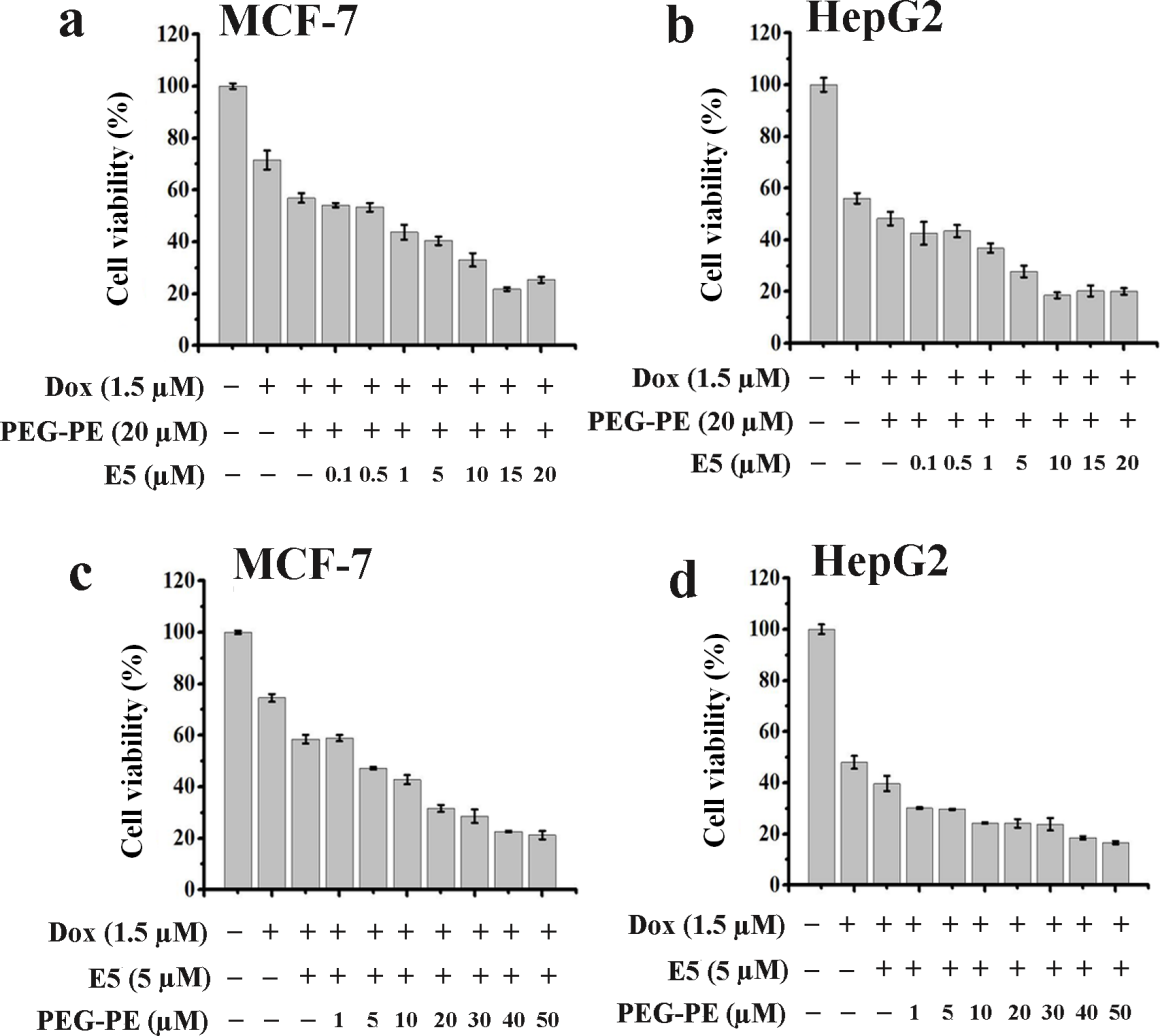
*In vitro* cytotoxicity assays of tumor cells after incubation with M-E5-Dox. Cell viabilities of (**a & c)** MCF-7 and (**b & d)** HepG2 tumor cells, assessed by MTS, after incubation with E5 (0.1–20 μM) and PEG-PE (1–50 μM) for 48 h at 37°C.

In addition, the cytotoxicities of MCF-7 and HepG2 tumor cells by treatment with various combinations of drugs were evaluated using MTS assay. As shown in Fig 6C, the IC_50_ of MCF-7 tumor cells by treatment with free Dox, M-Dox, AMD3100 + Dox, E5 + Dox, and M-E5-Dox for 48 h were 2.53, 1.21, 1.49, 0.98, and 0.57 μM, respectively. Meanwhile, the IC_50_ of HepG2 tumor cells by treatment with free Dox, M-Dox, AMD3100 + Dox, E5 + Dox, and M-E5-Dox for 48 h were 2.61, 0.98, 1.60, 1.08, and 0.79 μM, respectively (Fig 6D). These results indicated that the concentration of M-E5-Dox that caused 50% of cells death was much lower than that of free Dox, M-Dox, AMD3100 + Dox, E5 + Dox, implying that M-E5 played an important role in enhancing the sensitivity of MCF-7 and HepG2 tumor cells for Dox. Furthermore, we incubated MCF-7 and HepG2 tumor cells with M-E5-Dox at a fix concentration of Dox (1.5 μM) and with the increasing concentrations of E5 (0.1–20 μM) and PEG-PE (1–50 μM) for 48 h, and then evaluated the cell viability using MTS assay. As shown in Fig 7, both E5 and PEG-PE significantly increased the cytotoxicity of MCF-7 and HepG2 tumor cells in a concentration-dependent manner for Dox.

However, the caspase-3 activities of SKBR-3 tumor cells after free Dox **(**2 μM), M-Dox (Dox: 2 μM, PEG-PE: 20 μM) and M-E5-Dox (Dox: 2 μM, PEG-PE: 20 μM, and E5: 5 μM) treatments were increased by 3.1 ± 0.3, 4.1 ± 0.2 and 4.2 ± 0.5 folds compared with untreated cells, respectively (Panel a in S3 Fig). In addition, MTS results showed that there was no statistically significant difference of cytotoxicity between M-Dox and M-E5-Dox group *in vitro* (Panel b in S3 Fig). The results suggested that E5 could increase the sensitivity of CXCR4-overexpressing tumor cells for chemotherapeutic drugs via blocking CXCR4/CXCL12 axis, but didn’t show this effect in CXCR4-lowexpressing tumor cells.

**Fig 8:**
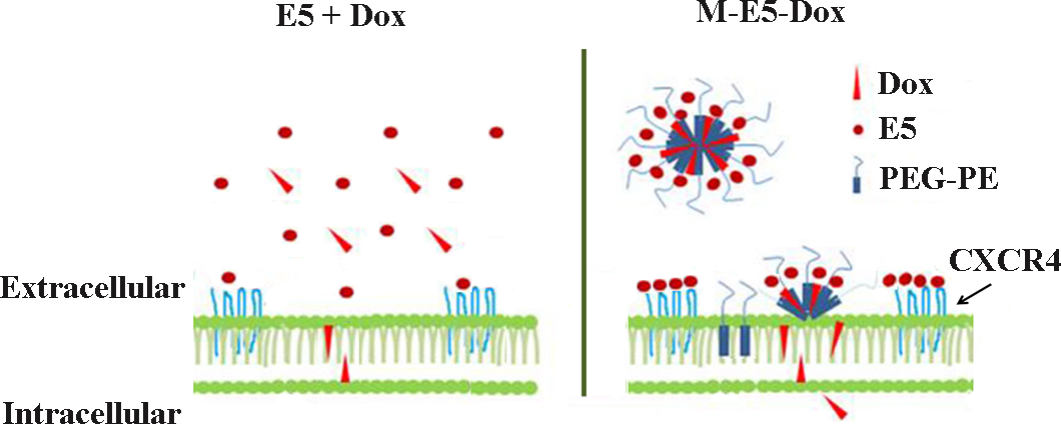
Schematic illustration of the interactions of “E5 + Dox” and “M-E5-Dox” with the tumor cell membranes.

Schematic illustration in Fig 8 showed that the possible mechanism of how M-E5 increases the sensitivity of CXCR4-overexpressing tumor cells for Dox. In the absence of PEG-PE micelle, E5 and Dox were dispersed evenly in the culture medium, and only a few molecules of E5 could target CXCR4 on the cell membrane and Dox could transport across the cell membrane. In another case of M-E5-Dox, PEG-PE micelle was capable of decreasing the concentrations of E5 and Dox in the culture medium, led to preferential adsorption and accumulation of E5 and Dox on the cell membrane. Drug-loaded PEG-PE micelle disassembled and released their payloads at the cell membrane. The disassembled E5 from M-E5-Dox could interact with CXCR4 on the cell membrane and disrupt the CXCR4/CXCL12 axis, while the disassembled Dox and PEG-PE copolymers could transport across the cell membrane into the cell by separate pathways, consistent with previous studies [21]. E5 enhanced the sensitivity of tumor cells for Dox by down-regulating the phosphorylation level of Akt, Erk and p38/MAPK proteins, which was resulted from the antagonistic effect of E5 on CXCR4/CXCL12 axis.

A growing number of studies have been reported that polymeric micelles comprised of PEG-PE have presented special interests because this carrier has some advantages [37–39]. The chemotherapeutic drug loaded PEG-PE micelles exhibited increased cytotoxicity *in vitro* and enhanced antitumor activity *in vivo* with low systemic toxicity, which has important clinical applications for cancer therapies [19–21,26,27]. This work is the first report about PEG-PE micelle being a promising drug delivery system for anti-tumor peptides. These results suggested M-E5 could inhibit the migration of CXCR4-overexpressing tumor cells mediated by CXCL12 and enhance the sensitivity of tumor cells for Dox *in vitro*. The antitumor effect of M-E5-Dox *in vivo* should be carefully addressed in the future to improve the application of M-E5-Dox as a potential therapeutic agent. As peptides are promising drug candidates for clinical application, the ability of PEG-PE micelle to modulate the bioactivity of peptides *in vivo* and *in vitro* opens up new avenues for peptide-based drug discovery.

## Conclusions

In this work, we have demonstrated that PEG-PE micelle could encapsulate a peptide antagonist E5 and improve E5 targeting efficiency for CXCR4 by accumulating E5 on the cell membrane. M-E5 could inhibit the migration of CXCR4-overexpressing MCF-7 and HepG2 tumor cells and enhance the sensitivity of tumor cells for Dox *in vitro*. CXCR4 is highly expressed in at least 22 various types of human malignant solid tumors, and PEG-PE copolymers are approved by FDA as a stealth material for Doxil^®^ that is a liposomal formulation of doxorubicin. Therefore, M-E5 is expected to be a potential therapeutic agent to improve the clinical benefits in current therapies used for solid tumors.

## Supporting information

S1 Fig CXCR4 expression levels over tumor cells. **(a**) CXCR4 expression levels over MCF-7, HepG2, SKBR-3, Panc-1, PC-3 and Hela cells were measured by flow cytometer using PE mouse anti-human CXCR4 and PE mouse IgG2a antibodies. (**b**) Percentages of CXCR4 expression levels. (**c**) Confocal images of CXCR4 expression levels on MCF-7 and HepG2 tumor cells.
(TIF)

S2 Fig The binding assays of E5 in the absence and presence of PEG-PE micelles with tumor cells. **(a**) Concentration- and (**b**) incubation time-dependent binding assays of E5 and M-E5 with SKBR-3 tumor cells. (**c**) Confocal images of SKBR-3 tumor cells after incubation with FITC-E5 (5 μM) in the absence and presence of PEG-PE micelles (20 μM) for 2 h at 37°C.
(TIF)

S3 Fig *In vitro* cytotoxicity assays of SKBR-3 tumor cells. **(a**) Effects of free Dox **(**2 μM), M-Dox (Dox: 2 μM, PEG-PE: 20 μM) and M-E5-Dox (Dox: 2 μM, PEG-PE: 20 μM, and E5: 5 μM) on the caspase-3 activities of SKBR-3 tumor cells after 24 h treatment at 37°C. (**b**) Cell viability of SKBR-3 tumor cells, assessed by MTS, after incubation with free Dox, M-Dox, AMD3100 + Dox, E5 + Dox, and M-E5-Dox for 48 h at 37°C. Data are presented as mean ± SD (*n* = 3). The * represents significant difference between two groups (*p < 0.05, **p < 0.01).
(TIF)

S4 Fig *In vitro* cytotoxicity assays of tumor cells after incubation with AMD3100, E5 and PEG-PE. Cell viabilities of (**a & c & e)** MCF-7 and (**b & d & f)** HepG2 tumor cells, assessed by MTS, after incubation with AMD3100 (0–200 μM), E5 (0–100 μM) and PEG-PE (0–200 μM) for 48 h at 37°C.
(TIF)

S1 Table Primers sequence used in present study. (DOCX)
