# Supplementary figures and images for: Anti-tumor activity of nanomicelles encapsulating CXCR4 peptide antagonist E5
Source: PLoS One. 2017 Aug 9;12(8):e0182697. doi: 10.1371/journal.pone.0182697 (PMC5549986; doi:10.1371/journal.pone.0182697)

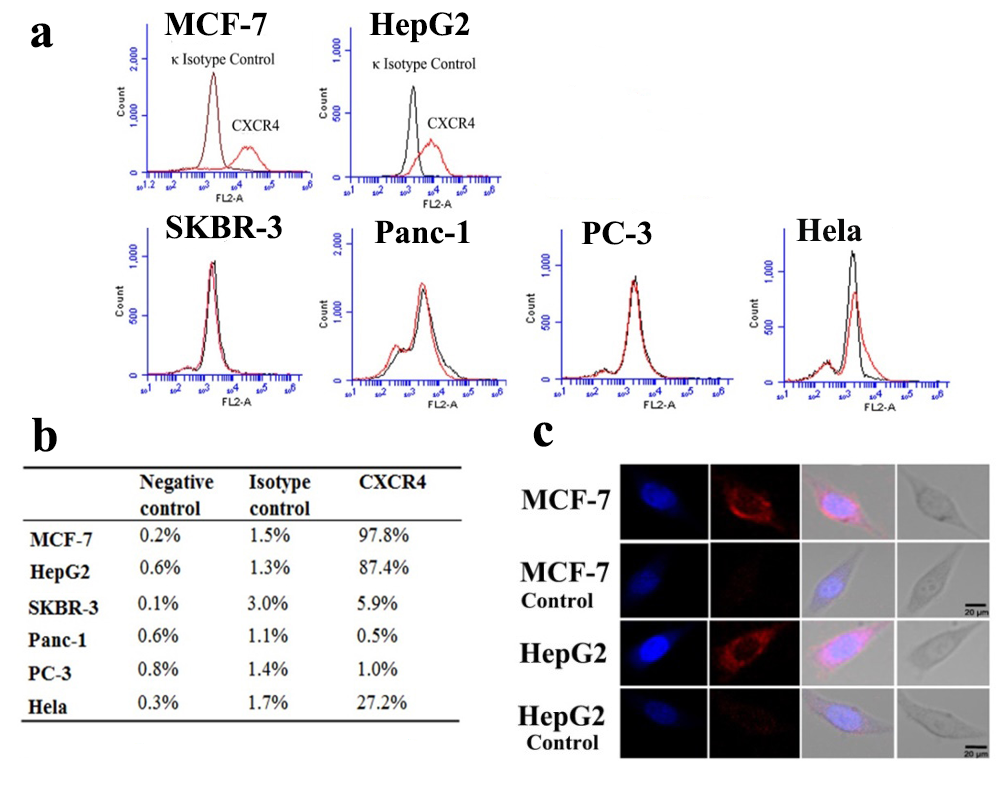

Supplement: S1 Fig — (a) CXCR4 expression levels over MCF-7, HepG2, SKBR-3, Panc-1, PC-3 and Hela cells were measured by flow cytometer using PE mouse anti-human CXCR4 and PE mouse IgG2a antibodies. (b) Percentages of CXCR4 expression levels. (c) Confocal images of CXCR4 expression levels on MCF-7 and HepG2 tumor cells. (TIF) [file pone.0182697.s001.tif]

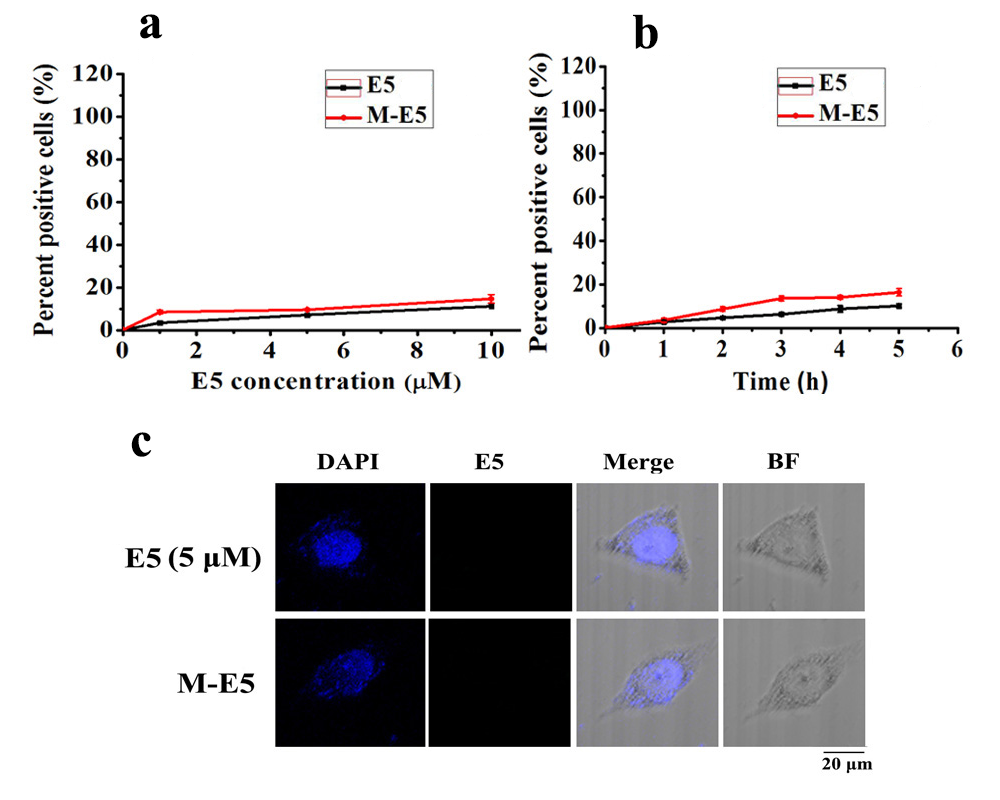

Supplement: S2 Fig — (a) Concentration- and (b) incubation time-dependent binding assays of E5 and M-E5 with SKBR-3 tumor cells. (c) Confocal images of SKBR-3 tumor cells after incubation with FITC-E5 (5 μM) in the absence and presence of PEG-PE micelles (20 μM) for 2 h at 37°C. (TIF) [file pone.0182697.s002.tif]

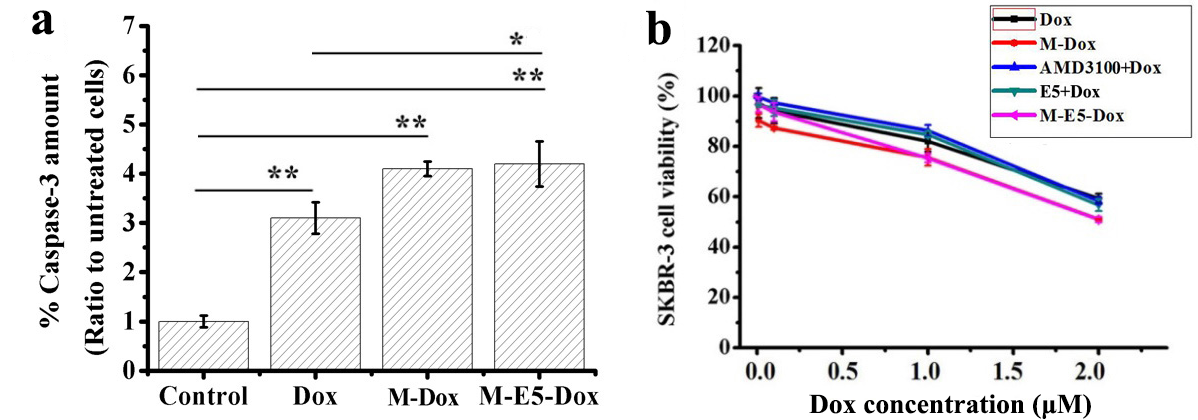

Supplement: S3 Fig — (a) Effects of free Dox (2 μM), M-Dox (Dox: 2 μM, PEG-PE: 20 μM) and M-E5-Dox (Dox: 2 μM, PEG-PE: 20 μM, and E5: 5 μM) on the caspase-3 activities of SKBR-3 tumor cells after 24 h treatment at 37°C. (b) Cell viability of SKBR-3 tumor cells, assessed by MTS, after incubation with free Dox, M-Dox, AMD3100 + Dox, E5 + Dox, and M-E5-Dox for 48 h at 37°C. Data are presented as mean ± SD (n = 3). The * represents significant difference between two groups (*p < 0.05, **p < 0.01). (TIF) [file pone.0182697.s003.tif]

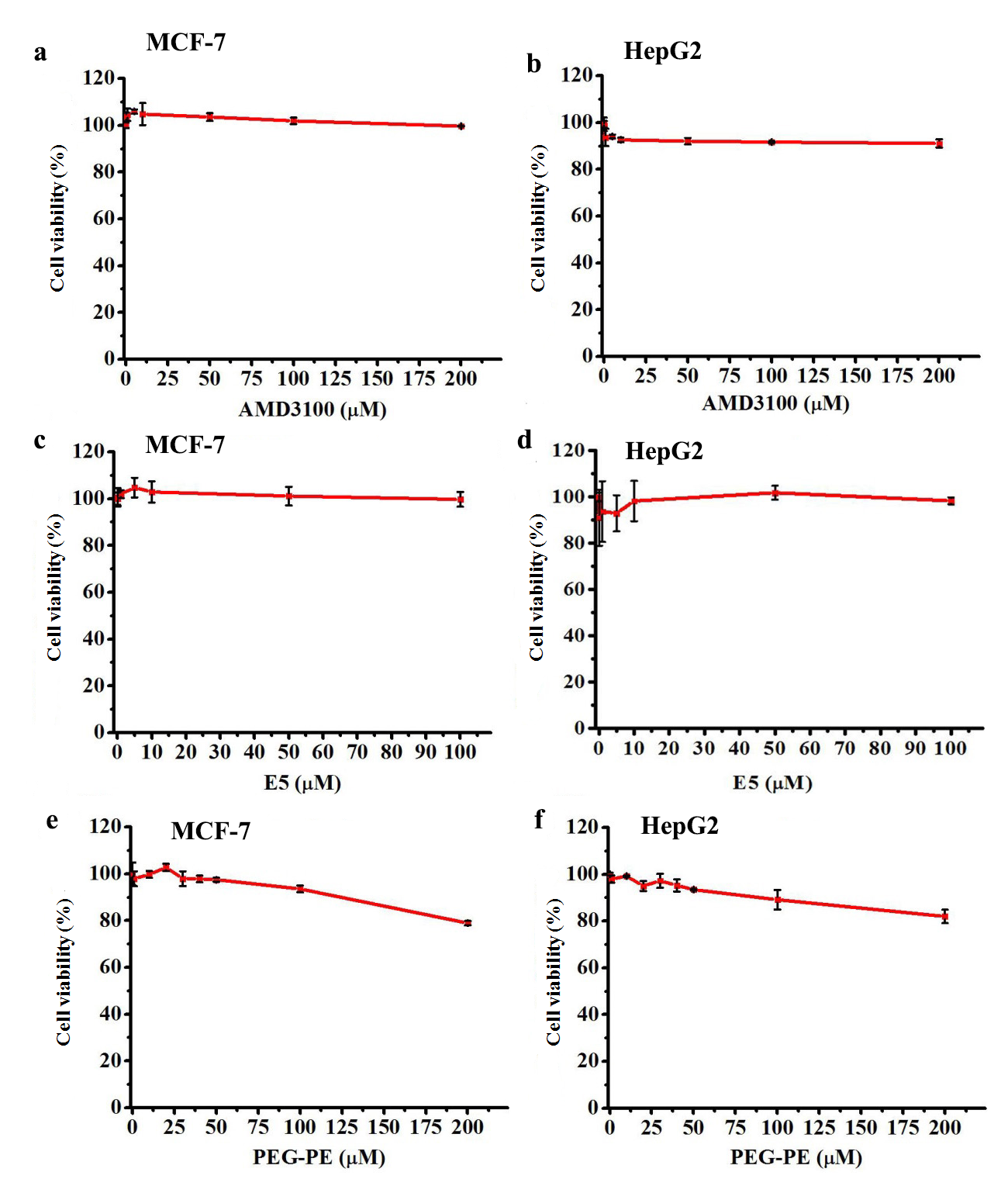

Supplement: S4 Fig — Cell viabilities of (a & c & e) MCF-7 and (b & d & f) HepG2 tumor cells, assessed by MTS, after incubation with AMD3100 (0–200 μM), E5 (0–100 μM) and PEG-PE (0–200 μM) for 48 h at 37°C. (TIF) [file pone.0182697.s004.tif]
