# Supplementary material for: Anti-tumor activity of nanomicelles encapsulating CXCR4 peptide antagonist E5
Source: PLoS One. 2017 Aug 9;12(8):e0182697. doi: 10.1371/journal.pone.0182697 (PMC5549986; doi:10.1371/journal.pone.0182697)
Supplement: S1 Table — (DOCX) [file pone.0182697.s005.docx]

**Table**

**S1 Table.**

| Primers  (human) | Sequence (5’– 3’) | |
| --- | --- | --- |
|  | Forward primer | Reverse primer |
| Vimentin | GAACGCCAGATGCGTGAAATG | CCAGAGGGAGTGAATCCAGATTA |
| N-cadherin | GAGGAGTCAGTGAAGGAGTCA | GGCAAGTTGATTGGAGGGATG |
| MMP2 | ACCCTCAGAGCCACCCCTAA | AGCCAGCAGTGAAAAGCCAG |
| MMP9 | TCCCTGGAGACCTGAGAACC | CGGCAAGTCTTCCGAGTAGTT |
| GAPDH | GAGAAGGCTGGGGCTCATTT | AGTGATGGCATGGACTGTGG |
